# Effects of Individualized Aerobic Exercise Training on Physical Activity and Health-Related Physical Fitness among Middle-Aged and Older Adults with Multimorbidity: A Randomized Controlled Trial

**DOI:** 10.3390/ijerph18010101

**Published:** 2020-12-25

**Authors:** Yi-Pang Lo, Shang-Lin Chiang, Chia-Huei Lin, Hung-Chang Liu, Li-Chi Chiang

**Affiliations:** 1 Graduate Institute of Medical Sciences, National Defense Medical Center, Taipei 11490, Taiwan; 808010023@mail.ndmctsgh.edu.tw; 2 Department of Nursing, Tri-Service General Hospital SongShan Branch, Taipei 10581, Taiwan; andyy520@mail.ndmctsgh.edu.tw; 3 School of Medicine, National Defense Medical Center, Taipei 11490, Taiwan; andyyy520@yahoo.com.tw; 4 Department of Physical Medicine and Rehabilitation, Tri-Service General Hospital, Taipei 11490, Taiwan; bryant480@gmail.com; 5 School of Nursing, National Defense Medical Center, Taipei 11490, Taiwan; 6 School of Nursing, China Medical University, Taichung 40402, Taiwan

**Keywords:** middle-aged, older adult, individualized, aerobic exercise, multimorbidity

## Abstract

The presence of multimorbidity in middle-aged and older adults, which reduces their physical activity and quality of life, is a global health challenge. Exercise is one of the most important health behaviors that individuals can engage in. Previous studies have revealed that aerobic exercise training is beneficial for healthy middle-aged and older adults and those with various chronic diseases, but few studies have designed individualized aerobic exercise training for individuals with multimorbidity. Although individuals with multimorbidity are considerably less adherent to physical activity interventions, telephone-based motivational interviewing may help in strengthening motivation and promoting behavioral change for increasing physical activity and health-related physical fitness. This study aimed to examine whether a 12-week individualized aerobic exercise training in a rehabilitation center combined with telephone-based motivational interviewing is effective in promoting physical activity and health-related physical fitness among middle-aged and older adults with multimorbidity. A randomized controlled trial was conducted. Forty-three participants (aged > 40) were recruited and randomly assigned to the intervention group, comparison group, or control group. The participants’ physical activity and health-related physical fitness were assessed at baseline and at 12 weeks. The results indicated that after individualized aerobic exercise training combined with telephone-based motivational interviewing, the participants reported increased total physical activity (*F*_in_ = 481.3*, p* = 0.011), vigorous-intensity physical activity (*F*_in_ *= 298.9, p* = 0.007), dominant and nondominant hand grip (kg) *(F*_in_ = 1.96, *p* = 0.019; *F*_in_ = 2.19, *p* = 0.027, respectively*)*, FEV_1_/FVC *(F*_in_ = 0.045, *p* = 0.043*)*, VO_2_ max (ml/kg/min) *(F*_in_ = 5.30, *p* = 0.001*)*, VO_2_ max predicted (%) *(F*_in_ = 21.6*, p* = 0.001*)*, work (watts) *(F*_in_ = 22.5, *p* = 0.001*)*, and anaerobic threshold (L/min) *(F*_in_ = 0.165, *p* = 0.011*)*. Twelve weeks of individualized aerobic exercise training in the rehabilitation center combined with telephone-based motivational interviewing can increase the total physical activity, vigorous physical activity, and cardiorespiratory fitness of middle-aged and older adults with multimorbidity.

## 1. Introduction

Multimorbidity is defined as the individual coexistence of two or more chronic diseases/illness conditions [1,2,3]. The World Health Organization (WHO) has noted that chronic diseases cause over 38 million deaths worldwide every year and are the leading cause of human death [4]. As the global population continues to age, increasing numbers of middle-aged and older adults will be affected by multimorbidity. It is also believed that multimorbidity will become the most common type of disease requiring treatment by medical care personnel, and such patients will become the primary clients of health care services; consequently, integrated health care will be necessary [5]. Approximately 42.8% of adults aged 45–64 years old and 73.1% of adults over 65 suffer from multimorbidity in the United States, and the proportion of people suffering from multimorbidity is estimated to gradually increase by more than 1% every year by 2030 [6]. Middle-aged and older adults with multimorbidity have been demonstrated to require a longer hospital stay, have more postoperative complications, have higher medical care costs and mortality, have higher degrees of psychological distress, have poor sleep quality, and have a poor quality of life [7,8,9].

Physical inactivity and poor health-related physical fitness are major risk factors for multimorbidity and increase the risk of death by 20–30% [4,10]. Increasing physical activity and good health-related physical fitness are goals for the healthcare of patients with multimorbidity. Previous evidence showed that the benefits of individualized aerobic exercise training or therapy could be beneficial for not only healthy middle-aged and older adults [11], patients with hemodialysis [12], cancer [13], but also of those with other chronic diseases [14]. Based on a systematic review, for patients with multimorbidity, exercise interventions could improve physical function, reduce depression and anxiety symptoms, and improve health-related quality of life [15]. However, these studies rarely discussed the outcomes of physical activity (PA) and health-related physical fitness.

Aerobic exercise is safer and can lower the susceptibility to certain preventable chronic diseases, decrease mortality, and improve health in middle-aged and older adults [15]. Aerobic exercise training should be individualized and tailored to the physiological and psychological status of each individual with multimorbidity [16].

Individualized aerobic exercise training is a recommended physical activity program designed in a systematic and individualized manner in terms of frequency, intensity, time, type, volume, and progression, known as the FITT-VP principle [17]. In individualized aerobic exercise training prescriptions, an incremental cardiopulmonary exercise test (CPET) is the gold standard to assess cardiorespiratory fitness, to determine maximal oxygen uptake (VO_2_ max) levels at different aerobic exercise training intensities, and to integrate and evaluate cardiovascular, respiratory, skeletal muscle, and neuromuscular responses to exercise [17,18,19]. However, few studies have evaluated individualized aerobic exercise training for patients with multimorbidity. The principles of exercise in people with multimorbidity include a rigorous assessment of health status, adapting the exercises to multimorbid condition, applying and integrating behavior change techniques into the exercise plan, and clinical reasoning to support the application of exercise by health professionals [20].

Aerobic training should be continued for as a long-term management to sustain this beneficial effect. However, patients with multimorbidity are significantly less adherent to exercise interventions than healthy individuals due to many barriers (e.g., disease-specific symptoms, fatigue, and lack of time) to exercise [21]. Motivational interviewing, which is a collaborative, patient-centered counseling approach, may be used to strengthen motivation and promote behavioral change among middle-aged and older adults in a community through open-ended discussions [22]. This approach is widely used in clinical care including the promotion of smoking cessation [23], type 2 diabetes self-management [24], and physical activity promotion in cancer survivors [25]. According to a systematic review and meta-analysis, motivational interviewing may support individuals to modify their cardiovascular risk through lifestyle modification [26]. Telephone-based motivational interviewing is also beneficial because it not only improves self-efficacy for physical activity and increases caloric expenditures from physical activity in middle-aged adults over 55 years [27], but also increases physical activity and reduces metabolic risk [28].

Therefore, this randomized controlled trial aimed to examine the differences of effectiveness of a 12-week individualized aerobic exercise training in a rehabilitation center combined with telephone-based motivational interviewing, only telephone-based motivational interviewing, and usual care on the physical activity and health-related physical fitness among middle-aged and older adults with multimorbidity.

## 2. Materials and Methods

### 2.1. Study Design

A randomized controlled trial with three groups was created (registered number: ISRCTN44497123). All eligible middle-aged and older adults with multimorbidity were randomized by a research assistant to one of three groups: (1) the intervention group, which received 12 weeks of a multidisciplinary (including a physician, nurse and physiotherapist) individualized aerobic exercise training program in the rehabilitation center combined with telephone-based motivational interviewing; (2) the comparison group, which received 12 weeks of telephone-based motivational interviewing; and (3) the control group, which received 12 weeks of usual care. In this study, multimorbidity was defined as participants with two or more of the following high prevalence chronic diseases based on aging survey report in our country [29]: hypertension, hyperlipidemia, diabetes, stroke, cancer, heart disease, kidney disease, asthma, chronic obstructive pulmonary disease, osteoporosis, degenerative arthritis, gout, depression, schizophrenia, and bipolar disorder.

### 2.2. Participants

Eligible participants were recruited by a research assistant from a rehabilitation clinic at a medical center in Taipei city, Taiwan from November 2016 to February 2017. Those who agreed to join the study were referred to a rehabilitation/sports medicine physician for screening to determine whether they had contraindications for aerobic exercise training. The inclusion criteria were as follows: (1) over 40 years old with multimorbidity; (2) no cognitive impairment (Mini-Mental State Examination, MMSE > 24); (3) able to communicate in Mandarin; (4) provided informed consent and agreed to be randomized into one of three groups; and (5) current physical activity amount did not meet the WHO recommendations (<150 min of moderate-intensity physical activity/week or <75 min of vigorous-intensity physical activity/week). The exclusion criteria were as follows: (1) aerobic exercise training contraindications according to the updated American College of Sports Medicine (ACSM) recommendations for exercise pre-participation health screening [30] as demonstrated by a rehabilitation/sports medicine physician; (2) unable to tolerate moderate to vigorous aerobic exercise training due to impaired neurogenic/musculoskeletal conditions; (3) unable to cooperate with aerobic exercise training; (4) unable to walk without assistance; and (5) have been judged to be unsuitable for participation in this study by a rehabilitation/sports medicine physician for other reasons (e.g., the participant was too nervous to complete the questionnaire or health-related physical fitness assessment).

### 2.3. Intervention

The participants were randomly assigned to either the intervention group, the comparison group, or the control group after receiving an individual 20-min face-to-face counseling session and educational brochure about the importance of regular exercise, the benefits of aerobic exercise training for multimorbidity, and physical activity recommendations/general aerobic exercise prescriptions based on WHO/ACSM guidelines [10,17].

The intervention group received 12 weeks of individualized aerobic exercise training in a rehabilitation center combined with telephone-based motivational interviewing that consisted of: (1) The rehabilitation/sports medicine physician collected and evaluated the participants’ medical history and lifestyle (focused on PA information), discussed the gathered information with the participants, and then designed the individualized exercise prescription according to the ACSM’s exercise management for persons with chronic diseases and disabilities [31]. (2) a trained nurse (the first author) and physiotherapist who coached and supervised the participant through the cycle ergometer aerobic exercise training and individualized exercise prescription recommendations. Based on the graded exercise test data and ACSM guideline FITT-VP principles [17], the nurse and physiotherapist coached and supervised the cycle ergometer aerobic training according to the individual guidelines for frequency (3–5 sessions/week, varied from participant to participant), intensity (gradually increased from 50% heart rate reserve to 80% heart rate reserve according to the Karvonen method [32]), time (30–50 min/session, which consisted of a 5-min warm-up period, a 20- to 40-min main training period to reach the target heart rate, and a 5-min cool-down period), type (cycle ergometer), volume (>150 min of moderate-intensity physical activity/week or >75 min of vigorous-intensity physical activity/week), and progression; (3) a trained nurse who introduced the Borg scale with numbers from 6 to 20 to rate perceived exertion (RPE) [33], and the participants were asked to report their level of exertion every 5 min to help the physiotherapist assess whether they had reached the target exercise intensity (RPE range: 12–16) and their fitness level during the cycle ergometer aerobic exercise training; (4) the participants’ EKG, heart rate, blood pressure, and peripheral blood oxygen concentration were continuously monitored during the cycle ergometer aerobic exercise training to ensure participants’ safety; and (5) face-to-face motivational interviewing with a nurse once a week to set the goals for the next week’s training, motivate the participant to reach WHO physical activity recommendations and ask the participants how they felt about the training. (6) The telephone-based motivational interviewing protocol modified by our previous study [28] was delivered for 15–30 min once a week, and it focused on determining participants’ PA and exercise stage, providing essential information of exercise benefit, giving personal feedback or encouraging them to engage in exercise, setting reasonable and weekly exercise goals, monitoring their exercise behaviors, reminding them to reach the PA recommendation, and providing rewards for achieving the goals. Table 1 summarizes the 12-week individualized aerobic exercise training combined with telephone-based motivational interviewing content. This combined intervention ensured the fidelity of intervention delivery.

Meanwhile, the comparison group only received 12 weeks of telephone-based motivational interviewing. The control group received 12 weeks of usual care, which consisted of general recommendations for physical activity.

### 2.4. Outcome Measurement

The outcomes were measured at baseline and after 12 weeks in all participants. The participants completed self-reported questionnaires on demographic data, self-perceived health status questionnaires, and the International Physical Activity Questionnaire (IPAQ)-Chinese version short form. Objective measurements of health-related physical fitness parameters were performed by a well-trained research assistant.

The demographic questionnaires assessed age, gender, marital status, whether the participants had children, educational level, employment status, and number/type of chronic diseases. The self-perceived health status questionnaire consists of five items with a 5-point Likert scale, which scores from 5 to 25. The higher the score, the better the self-perceived health status. It has good content validity, construct validity, and internal consistency; thus, it has been widely used in several studies in Taiwan [34].

The IPAQ-Chinese version short form has well-established reliability and validity [35]. This seven-item instrument measures the minutes spent on physical activity including housework, transportation, leisure activity, and moderate-to-vigorous-intensity physical activity for the past seven days. The IPAQ scores, which are expressed as “MET-minutes/week,” were calculated based on the MET level (i.e., walking = 3.3 METs, moderate-intensity physical activity = 4.0 METs, vigorous-intensity physical activity = 8.0 METs) multiplied by the minutes of each activity per week [36].

Prior to the health-related physical fitness assessment, the participants were asked to avoid doing vigorous exercises and sleep at least 6–8 h a day, and avoid food, alcohol, and caffeine for at least 3 h prior. They should also wear appropriate clothing for the assessment. The assessments were conducted from 2:00 PM to 5:00 PM, and each participant was assessed for approximately 60 min. The assessment room was kept private, quiet, and well ventilated to make the participants feel comfortable. The health-related physical fitness assessment based on the ACSM’s health-related physical fitness assessment manual and exercise testing guidelines [17,19] was performed as follows: (1) Resting heart rate and blood pressure were measured by the JPN1 automatic blood pressure monitor (OMRON, Osaka, Japan). (2) Body height and weight were measured by the BW-2986VM automatic height and weight scale (NAGATA, Tainan, Taiwan), and the body mass index (BMI) was calculated to determine body composition. (3) Upper limb grip strength was measured using the TKK-5401 digital handgrip dynamometer (Matsuyoshi & Co., Tokyo, Japan) to assess muscular strength. During the measurement, the participants were asked to stand, flex their elbows at 90°, and squeeze the handgrip dynamometer using their dominant/nondominant hand thrice as hard as possible without holding the breath, and the highest measure (in kilograms) of the dominant/nondominant hand was recorded. (4) The 30-s sit-to-stand test was performed using a stopwatch and a chair with a straight back, a seat height of 43 cm, and no armrest to assess muscular endurance. The participants were asked to place their hands on the opposite shoulder crossed and repeat the standing and sitting positions within 30 s; the number of times the participants came to a full standing position was counted and recorded. (5) The chair sit-and-reach test was performed to assess flexibility. Here the participants were asked to sit on the edge of the chair, remained on foot flat on the floor, stretched the other leg forward with the knee straight, heel on the floor, and dorsiflexion of 90°. Next, the participants were instructed to inhale and then exhale while reaching the toes by bending at the hip, with the knees kept straight, and the reach was held for 2 s. The distance between the fingertips and the toes was measured. If the fingertips touched the toes, then the score was 0; otherwise, the distance between the fingers and the toes (a negative score) was measured, and if they overlapped, the distance of overlap was measured (a positive score). (6) Pulmonary function tests (Vmax Encore 29 exercise testing system, Viasys, Mettawa, IL, USA) were conducted under the supervision of a rehabilitation/sports medicine physician. (7) Pre-exercise testing was performed to identify potential absolute/relative contraindications (e.g., unstable angina, uncontrolled symptomatic heart failure etc.) to cardiopulmonary exercise testing, and the safety of the test was ensured by rehabilitation/sports medicine physician. (8) Finally, cardiopulmonary exercise testing (Vmax Encore 29 exercise testing system, Viasys, Mettawa, IL, USA) was performed to determine the maximal oxygen uptake (VO_2_ max), anaerobic threshold (AT), and work, etc., to assess cardiorespiratory fitness. Cycle ergometer-based (Corival, Lode, Groningen, The Netherlands) graded exercise test (GXT) was conducted using a ramp protocol by increasing the resistance (10 W/min) at a consistent speed of 60 revolutions/minute, supervised by a rehabilitation/sports medicine physician. The participants’ EKG, heart rate, blood pressure, and peripheral blood oxygen concentration were continuously monitored, and the Borg scale was used to evaluate the rate of perceived exertion; the measures assured the safety of the participants. After the health-related physical fitness assessment was completed, the participants rested and relaxed for 5–10 min in place and then left after the researcher confirmed that they felt no physical discomfort.

### 2.5. Sample Size

G*Power software (version 3.1.9.2, Universität Kiel, Kiel, Germany) was applied for estimating the sample size [37]. Based on using multivariate analysis of variance: repeat measures, within-between interaction with a medium effect size of 0.5 [38], α = 5%, a power of 80%, and three groups with two measurements, we calculated the sample size of 14 is necessary for each group.

### 2.6. Randomization and Blinding

The randomization sequence was generated using the random number function in Microsoft Excel (Microsoft Corp., Redmond, WA, USA). A block of 6 randomization was designed and stratified by age (<65 years old, ≥65 years old) and gender (male, female). The researchers involved in the data collection were blinded to the group allocation. Participant blinding was not possible due to the nature of the intervention conditions.

### 2.7. Ethical Consideration

Institutional review board approval (TSGHIRB: 1-105-05-156) was obtained from Tri-Service General Hospital in Taiwan. All participants gave written informed consent when they were invited to join the study and were assured that their participation was entirely voluntary and that they could withdraw at any time.

### 2.8. Statistical Methods

The statistical analysis was performed with SPSS Version 22.0 (IBM Corp., Armonk, NY, USA). Descriptive statistics including the means and standard deviations (SD) or number and percentages (%) for the study participants were presented. The Chi-square test or analysis of variance (ANOVA) were used to compare the pre-intervention differences among the three groups. Some variables were not normally distributed, so the Kruskal–Wallis method was used to test the homogeneity of continuous dependent variables with three groups.

The mixed model ANOVA is a combination of a between-unit ANOVA and a within-unit ANOVA, allowing for evaluating interaction effects regarding time (pre vs. post) x groups (all three groups included), is standard procedure when evaluating intervention effects in the field of exercise physiology [39,40]. For repeated measurements, the mixed model is better than the general linear model in dealing with missing data at follow-up [41].

Therefore, we used mixed-model ANOVA to estimate the intervention effects of the three groups based on significant interactions of group and time. The intent-to-treat analysis was used. The significance level was set at 0.05 for two-tailed tests, and *p* < 0.05 was considered statistically significant. The Bonferroni test was used for post-hoc comparison.

## 3. Results

### 3.1. Recruitment

**Figure 1:**
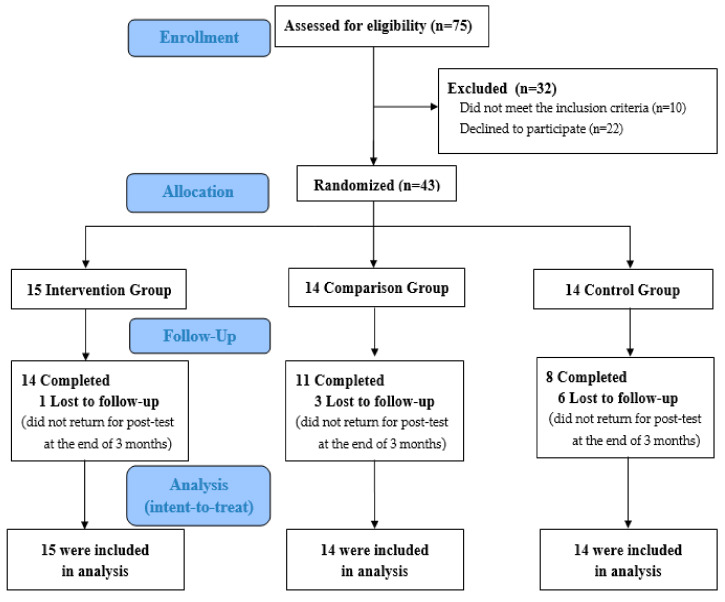
CONSORT study flow chart.

The participant CONSORT study flow chart is shown in Figure 1. From November 2016 to June 2017, 75 individuals were initially approached. After screening for eligibility, the remaining 43 participants were randomly assigned to either the intervention group (15 participants), the comparison group (14 participants), or the control group (14 participants). Of the 43 randomized participants, 33 (77%) completed all data collections, including 14 in the intervention group, 11 in the comparison group, and eight in the control group.

### 3.2. Baseline Characteristics of the Participants

The participants’ baseline characteristics are described in Table 2. The participants’ mean age was 64.4 ± 7.9 (years), 21 (48.8%) were male and average had 3.7 ± 1.1 chronic diseases. No statistically significant differences in the demographic characteristics, number of chronic diseases, self-perceived health status, physical activity amount, and health-related physical fitness were found between the three groups. The prevalence of each chronic disease among the participants was as follows: hypertension (*n* = 34, 79.1%), hyperlipidemia (*n* = 34, 79.1%), diabetes (*n* = 16, 37.2%), stroke (*n* = 1, 2.3%), cancer (*n* = 2, 4.7%), heart disease (*n* = 21, 48.8%), kidney disease (*n* = 3, 7.0%), asthma (*n* = 5, 11.6%), chronic obstructive pulmonary disease (*n* = 6, 14.0%), osteoporosis (*n* = 11, 25.6%), degenerative arthritis (*n* = 20, 46.5%), gout (*n* = 6, 14.0%), depression (*n* = 2, 4.7%), schizophrenia (*n* = 0, 0%), and bipolar disorder (*n* = 0, 0%).

### 3.3. Outcome Evaluation

The effectiveness of the different interventions on the PA amount and levels based on the mixed model analysis are shown in Table 3. The significant group x time interaction for PA revealed that middle-aged and older adults with multimorbidity in the intervention group had a greater increase in total PA (*F*_in_ = 481.3, *p* = 0.011) and vigorous-intensity PA (*F*_in_ = 298.9, *p* = 0.007) at 12 weeks than did participants in the control group. Moreover, we found that middle-aged and older adults with multimorbidity in the comparison group had a greater increase in walking PA (*F*_in_ = 505.7, *p* = 0.018) and a greater decrease in sedentary time (min/day) (*F*_in_ = −92.9, *p* = 0.025) compare to that of the control group.

The effectiveness of the different interventions on body composition, muscular strength, muscular endurance, and flexibility based on the mixed model analysis is shown in Table 4. The significant group × time interaction for body composition revealed that middle-aged and older adults with multimorbidity in the intervention group had a greater increase in weight (kg) and BMI (kg/m^2^) (*F*_in_ = 1.91, *p* = 0.028; *F*_in_ = 0.685, *p* = 0.030) at 12 weeks than did those in the comparison group. We also found that the significant group x time interaction for muscular strength revealed that middle-aged and older adults with multimorbidity in the intervention group had a greater increase in dominant hand grip (kg) and nondominant hand grip (kg) (*F*_in_ = 1.96, *p* = 0.019; *F*_in_ = 2.19, *p* = 0.027, respectively) at 12 weeks than did those in the control group. However, there were no statistically significant differences in muscular endurance and flexibility between the three groups.

The effectiveness of the different interventions on cardiorespiratory fitness based on the mixed model analysis are shown in Table 5. At 12 weeks, the FVC (L) and FEV_1_/FVC in three groups were significant change within pre- and post-test (*F*_w_ = 0.4, *p* = 0.011; *F*_w_ = −0.05, *p* = 0.044). The significant group × time interaction for cardiorespiratory fitness revealed that middle-aged and older adults with multimorbidity in the intervention group had greater increases in FEV_1_/FVC (*F*_in_ = 0.045, *p* = 0.043), VO_2_ max (ml/kg/min) (*F*_in_ = 5.30, *p* = 0.001), VO_2_ max predicted (%) (*F*_in_ = 21.6, *p* = 0.002), work (watts) (*F*_in_ = 22.5, *p* = 0.001), and AT (L/min) (*F*_in_ = 0.165, *p* = 0.011) at 12 weeks than participants in the control group. Moreover, the significant group × time interaction for cardiorespiratory fitness revealed that middle-aged and older adults with multimorbidity in the intervention group had greater increases in work (watts) (*F*_in_ = 21.7, *p* = 0.012) and AT (L/min) (*F*_in_ = 0.175, *p* = 0.004) at 12 weeks than participants in the comparison group.

## 4. Discussion

The results revealed that 12-week individualized aerobic exercise training combined with telephone-based motivational interviewing had significant beneficial effects on total PA, vigorous-intensity PA, muscular strength in dominant and nondominant hand grip, and several outcomes of cardiorespiratory fitness (FEV_1_/FVC, VO_2_ max, VO_2_ max predicted, work, and AT) among middle-aged and older adults with multimorbidity. Our results are consistent with those of previous studies, which reported that providing supervised, hospital-based aerobic exercise training results in positive effects on PA [42] and cardiorespiratory fitness [43,44,45]. Interestingly, increased muscular strength in dominant and nondominant hand grip may be one of the health benefits of cycle ergometer training [46].

As revealed in our results, middle-aged and older adults with multimorbidity in the intervention group exhibited increased total PA and vigorous-intensity PA and the comparison group also had significantly higher walking PA and significantly lower sedentary time at 12 weeks than the control group. A systematic review and meta-analysis reported the same situation wherein motivational interviewing had an effect in increasing the PA levels in people with chronic health conditions relative to the control groups (standardized mean differences = 0.19, 95% CI: 0.06–0.32, *p* = 0.004), but no significant effect on cardiorespiratory fitness and functional exercise capacity [47]. This finding may explain why the intervention group was still significantly better than the comparison group in some cardiopulmonary fitness variables (e.g., work and AT).

Although both intervention and comparison groups resulted in beneficial effects on PA, only the 12-week individualized aerobic exercise training combined with telephone-based motivational interviewing had a more significant effect on health-related physical fitness. These additional benefits highlight the importance of the combination of individualized aerobic exercise training and telephone-based motivational interviewing are consistent with those of previous studies, which reported that individuals engaged in more minutes of PA per week and were more likely to meet PA recommended levels/amount, more likely to maintain their exercise habits once after the hospital-based cardiac rehabilitation program finished [48], and the hospital-based cardiac rehabilitation program provided a catalyst to maintain PA and cardiopulmonary fitness in the 2 years following cardiac event [49].

In our study, the intervention group had no significant weight change after 12 weeks, consistent with the result in a previous study wherein 81 sedentary premenopausal women still did not lose body weight or fat mass after undergoing dual-energy x-ray absorptiometry before and after 12 weeks of supervised treadmill walking 3 days per week for 30 min at 70% of VO_2_ max [50]. Our study did not use bioelectrical impedance analysis instrument to gather fat mass and fat-free mass data; thus, we could not explain the reason behind the significant weight loss in the comparison group after 12 weeks and the significant difference between the intervention group and comparison group after 12 weeks.

Although self-report questionnaires may have recall/social desirability bias problems and tend to overestimate PA amount, they are the most common, convenient, and low-cost method of PA assessment [51,52]. Hence, we used a self-report questionnaire (IPAQ-Chinese version short form) to assess participants’ PA amount. In future studies, incorporating the accurate, reliable, and objective measures of PA using a pedometer or accelerometer is recommended.

This study has several unavoidable limitations including the relatively small sample size, the short follow up duration, and the limited human physiological parameters that were measured. Although the effectiveness of the aerobic exercise training can be evaluated after 12 weeks in most studies [15], we designed an intervention wherein we combined the training program with motivational interviewing; hence, follow-up on adherence to physical activity recommendations and behavioral change-related outcomes in a longer period (24–48 weeks) with more repeat measures is recommended [26]. Moreover, although we had evaluated the gold standard outcomes of the cardiorespiratory fitness, other parameters such as physical functioning and quality of life are also important to be addressed. Selection bias may have occurred because the sample participants were recruited from only one rehabilitation center and thus cannot be generalized to all types of multimorbidity patients in the hospital.

The novelty and strengths of this study are as follows: (1) we addressed the population with multimorbidity who will become the major clients of healthcare services in the near future, (2) we designed a multidisciplinary individualized aerobic exercise training program in a rehabilitation center in accordance with the ACSM’S guidelines and gold standard CPET to ensure the effectiveness of the intervention, (3) we combined the program with telephone-based motivational interviewing to reinforce the behavioral change and engage in exercise, (4) and we collected and evaluated the gold standard outcomes of the cardiorespiratory fitness that are seldom mentioned in this population.

Based on the study results, we recommend the combination of coached/supervised individualized aerobic exercise training with motivational interviewing following individualized exercise prescription recommendations to obtain the benefits of physical activity and improved health-related physical fitness among middle-aged and older adults with multimorbidity. Integrated aerobic exercise training program with multidisciplinary team can be used to initiate health promotion campaigns that revolve around patient-centered care. Furthermore, introducing resistance exercise training to middle-aged and older adults with multimorbidity would possibly further enhance their PA, health-related physical fitness, physical functioning, and quality of life.

## 5. Conclusions

To the best of our knowledge, this is the first 12-week individualized aerobic exercise training combined with telephone-based motivational interviewing randomized controlled trial among middle-aged and older adults with multimorbidity. A 12-week multidisciplinary individualized aerobic exercise training in the rehabilitation center combined with telephone-based motivational interviewing can increase total PA, vigorous-intensity PA, hand grip strength and cardiorespiratory fitness. A 12-week telephone-based motivational interviewing can also increase the amount of walking PA and can decrease sedentary time. Therefore, further research focused on larger samples and with a longer follow-up period to evaluate the efficacy/adherence of individualized aerobic exercise training combined with telephone-based motivational interviewing is recommended.

## Figures and Tables

**Table 1:** 12-week individualized aerobic exercise training combined with telephone-based motivational interviewing content.

Multidisciplinary Individualized Aerobic Exercise Training in the Rehabilitation Center (30–50 Min/Session)	
Rehabilitation/sports medicine physician	1. Collect and evaluate the participants’ medical history and lifestyle 2. Discuss with participants and then design the individualized exercise prescription
Physiotherapist	1. Coach the cycle ergometer aerobic training based on the FITT-VP principles 2. Assess the exercise intensity and fitness level of the participants
Trained nurse	1. Supervise the cycle ergometer aerobic training 2. Introduce the Borg scale 3. Conduct a face-to-face motivational interviewing to set the goals for the next week’s training, motivate the participant to reach WHO physical activity recommendations and ask them how they felt about the training
**Telephone-based motivational interviewing (15–30 min, once a week)**	
Four processes	Core content
1. Engaging 2. Focusing 3. Evoking 4. Planning	1. Determining the physical activity and exercise stage of the participants 2. Providing essential information on exercise benefit 3. Giving personal feedback or encouraging the participants to engage in exercise 4. Setting reasonable and weekly exercise goals for the participants 5. Monitoring the participants’ exercise behavior 6. Reminding the participants to reach the physical activity recommendation 7. Providing rewards for achieving the goals

**Table 2:** Comparison of baseline characteristics including demographics, number of chronic diseases, self-perceived health status, physical activity amount, and health-related physical fitness.

Characteristics	Intervention	Comparison	Control	F*/x*^2^	*p*
	(*n* = 15)	(*n* = 14)	(*n* = 14)		
**Age**				0.15	0.927
40–64 (years)	6 (40.0)	6 (42.9)	5 (35.7)		
≥65 (years)	9 (60.0)	8 (57.1)	9 (64.3)		
**Gender**				0.04	0.979
Male	7 (46.7)	7 (50.0)	7 (50.0)		
Female	8 (53.3)	7 (50.0)	7 (50.0)		
**Marital status**				2.47	0.650
Married	11 (73.3)	12 (85.7)	10 (71.4)		
Single	1 (6.7)	0 (0)	2 (14.3)		
Divorced/widowed	3 (20.0)	2 (14.3)	2 (14.3)		
**Have children**				0.61	0.736
Yes	14 (93.3)	13 (92.9)	12 (85.7)		
No	1 (6.7)	1 (7.1)	2 (14.3)		
**Educational level**				8.51	0.385
Elementary	1 (6.7)	2 (14.3)	3 (21.4)		
Junior high school	0 (0)	3 (21.4)	2 (14.3)		
Senior high school	9 (60.0)	4 (28.6)	3 (21.4)		
College/university	4 (26.7)	3 (21.4)	5 (35.7)		
Graduated	1 (6.7)	2 (14.3)	1 (7.1)		
**Currently employed**				3.55	0.169
Yes	7 (46.7)	2 (14.3)	5 (35.7)		
No	8 (53.3)	12 (85.7)	9 (64.3)		
**Number of chronic diseases**	3.7 ± 1.2	3.6 ± 0.9	3.8 ± 1.1	0.15	0.861
**Self-perceived health status**	15.5 ± 2.2	14.6 ± 3.0	15.3 ± 2.6	0.44	0.650
**Physical activity amount**					
**PA amount (MET-min/week)**					
Total PA	1132.1 ± 681.1	1321.3 ± 873.2	989.1 ± 522.5	0.78	0.465
Vigorous PA	64.0 ± 179.4	34.3 ± 128.3	45.7 ± 132.1	0.15	0.864
Moderate PA	333.3 ± 421.3	545.7 ± 661.9	238.6 ± 265.2	1.53	0.230
Walking PA	734.8 ± 502.4	741.3 ± 455.2	704.8 ± 229.9	0.03	0.970
**Sedentary time (min/day)**	350.7 ± 111.3	360.0 ± 111.0	360.0 ± 93.4	0.03	0.968
**Health-related physical fitness**					
**Body composition**					
Weight (kg)	65.7 ± 11.2	71.2 ± 17.8	68.5 ± 8.9	0.66	0.525
BMI (kg/m^2^)	24.4 ± 2.9	26.2 ± 4.7	25.6 ± 3.4	0.87	0.429
**Muscular strength**					
Dominant hand grip (kg)	28.3 ± 8.7	27.4 ± 8.8	27.6 ± 8.0	0.04	0.957
Nondominant hand grip (kg)	23.8 ± 8.5	24.8 ± 7.9	25.3 ± 7.5	0.13	0.878
**Muscular endurance**					
30 s sit-to-stand test (no.)	13.3 ± 4.5	14.5 ± 2.7	12.9 ± 1.4	0.92	0.408
**Flexibility**					
Chair sit-and-reach test (cm)	−10.0 ± 9.3	−6.0 ± 11.8	−7.6 ± 12.2	0.47	0.626
**Cardiorespiratory fitness**					
FVC (L)	3.3 ± 0.7	3.4 ± 0.9	3.5 ± 1.2	0.19	0.831
FEV_1_ (L)	2.6 ± 0.6	2.7 ± 0.7	2.8 ± 0.9	0.31	0.732
FEV_1_/FVC	0.8 ± 0.1	0.8 ± 0.1	0.8 ± 0.1	0.17	0.847
VO_2_ max (ml/kg/min)	22.0 ± 5.4	23.3 ± 5.9	24.7 ± 6.0	0.79	0.459
VO_2_ max predicted (%)	88.0 ± 22.0	95.9 ± 21.1	104.0 ± 25.2	1.78	0.182
Work (watts)	73.5 ± 17.9	90.6 ± 35.7	89.1 ± 24.7	1.82	0.175
AT (L/min)	0.8 ± 0.2	0.8 ± 0.3	0.8 ± 0.2	0.27	0.767

Note. The data are presented as the mean ± SD or number and percentages (%). *p* values are from the Chi-square test or ANOVA as appropriate. Some variables were not normally distributed, so the Kruskal–Wallis method was used to test the homogeneity of continuous dependent variables with three groups. PA, physical activity; BMI, body mass index; FVC, forced vital capacity; FEV_1_, forced expiratory volume in one second; VO_2_ max, maximal oxygen uptake; AT, anaerobic threshold.

**Table 3:** The effectiveness of individualized aerobic exercise training combined with telephone-based motivational interviewing on physical activity amount based on mixed model analysis.

Physical Activity Amount	Intervention		Comparison		Control		Between-Groups	Within-Times	Group (Intervention)			Group (Intervention)			Group (Comparison)		
	(*n* = 15)		(*n* = 14)		(*n* = 14)		*F*_b_ (*p*) ^a^	*F*_w_ (*p*) ^b^	×Time, *F*_in_^c^			×Time, *F*_in_^c^			×Time, *F*_in_^c^		
	Mean (SD)		Mean (SD)		Mean (SD)				*F*	SE	*p* ^d^	*F*	SE	*p* ^e^	*F*	SE	*p* ^d^
**Total PA**							143.1 (0.530)	63.7 (0.625)	481.3	189.1	0.011 *	−55.4	305.7	0.858	553.5	294.3	0.077
Baseline	1132 (681.1)		1321 (873.2)		989 (522.5)												
12 weeks	1692 (624.1)		1919 (803.5)		1068 (780.5)												
**Vigorous-intensity PA**							18.3 (0.755)	5.4 (0.947)	298.9	90.9	0.007 *	140.5	123.6	0.268	162.1	83.8	0.079
Baseline	64 (179.4)		34 (128.3)		46 (132.1)												
12 weeks	340 (329.8)		198 (300.3)		51 (160.0)												
**Moderate-intensity PA**							94.8 (0.573)	202.5 (0.187)	−66.0	171.8	0.707	51.3	187.4	0.787	−117.3	230.0	0.615
Baseline	333 (421.3)		546 (661.9)		239 (265.2)												
12 weeks	447 (261.7)		598 (425.2)		436 (378.2)												
**Walking PA**							30.0 (0.877)	−117.6 (0.488)	287.4	194.4	0.161	−218.4	253.6	0.398	505.7	189.5	0.018 *
Baseline	735 (502.4)		741 (455.2)		705 (229.9)												
12 weeks	897 (665.1)		1128 (765.2)		586 (378.2)												
**Sedentary time** **(min/day)**							−9.3 (0.824)	−12.7 (0.693)	−52.5	37.2	0.179	40.4	46.1	0.391	−92.9	37.7	0.025 *
Baseline	351 (111.3)		360 (111.0)		360 (93.4)												
12 weeks	290 (76.2)		243 (101.0)		348 (108.8)												

Note: PA, physical activity. ^a^ *F*_b_: The *F* value of between groups comparison. ^b^ *F***_w_**: The *F* value of within pre- and post-test. ^c^ *F*_in_: The *F* value of the interaction of between groups and within pre- and post-test. ^d^ Reference group: Group (Control) × Time. ^e^ Reference group: Group (Comparison) × Time. * *p* < 0.05. The Bonferroni test was used for post-hoc comparison.

**Table 4:** The effectiveness of individualized aerobic exercise training combined with telephone-based motivational interviewing on body composition, muscular strength, muscular endurance, and flexibility based on mixed model analysis.

Health-Related Physical Fitness	Intervention		Comparison		Control		Between-Groups	Within-Times	Group (Intervention)			Group (Intervention)			Group (Comparison)		
	(*n* = 15)		(*n* = 14)		(*n* = 14)		*F*_b_ (*p*) ^a^	*F*_w_ (*p*) ^b^	×Time, *F*_in_^c^			×Time, *F*_in_^c^			×Time, *F*_in_^c^		
	Mean (SD)		Mean (SD)		Mean (SD)				*F*	SE	*p* ^d^	*F*	SE	*p* ^e^	*F*	SE	*p* ^d^
**Weight (kg)**							−2.9 (0.563)	0.3 (0.660)	0.576	0.896	0.529	1.91	0.808	0.028 *	−1.33	0.801	0.115
Baseline	65.7 (11.2)		71.2 (17.8)		68.5 (8.9)												
12 weeks	66.5 (10.9)		70.3 (19.2)		68.8 (6.6)												
**Body mass index (kg/m^2^** **)**							−1.2 (0.403)	0.1 (0.644)	0.108	0.331	0.748	0.685	0.295	0.030 *	−0.578	0.294	0.069
Baseline	24.4 (2.9)		26.2 (4.7)		25.6 (3.4)												
12 weeks	24.7 (3.4)		25.8 (5.1)		25.7 (2.4)												
**Dominant hand grip (kg)**							0.6 (0.847)	−1.0 (0.238)	1.96	0.770	0.019 *	1.13	1.18	0.960	0.834	1.18	0.489
Baseline	28.3 (8.7)		27.4 (8.8)		27.6 (8.0)												
12 weeks	29.2 (8.2)		27.2 (10.1)		26.5 (7.9)												
**Nondominant hand grip (kg)**							−1.5 (0.620)	−1.7 (0.109)	2.19	0.910	0.027 *	1.42	1.37	0.316	0.775	1.35	0.575
Baseline	23.8 (8.5)		24.8 (7.9)		25.3 (7.5)												
12 weeks	24.3 (7.3)		23.7 (8.2)		23.5 (8.2)												
**30 s sit-to-stand test (no.)**							0.4 (0.726)	−0.6 (0.474)	1.02	0.589	0.101	0.04	1.073	0.970	0.979	0.931	0.314
Baseline	13.3 (4.5)		14.5 (2.7)		12.9 (1.4)												
12 weeks	14.0 (3.2)		15.0 (3.8)		12.5 (1.6)												

Note: Body mass index is the body weight (kg) divided by height squared (m). ^a^ *F*_b_: The *F* value of between groups comparison. ^b^ *F***_w_**: The *F* value of within pre- and post-test. ^c^ *F*_in_: The *F* value of the interaction of between groups and within pre- and post-test. ^d^ Reference group: Group (Control) × Time. ^e^ Reference group: Group (Comparison) × Time. * *p* < 0.05. The Bonferroni test was used for post-hoc comparison.

**Table 5:** The effectiveness of individualized aerobic exercise training combined with telephone-based motivational interviewing on cardiorespiratory fitness based on mixed model analysis.

Cardiorespiratory Fitness	Intervention		Comparison		Control		Between-Groups	Within-Times	Group (Intervention)			Group (Intervention)			Group (Comparison)		
	(*n* = 15)		(*n* = 14)		(*n* = 14)		*F*_b_ (*p*) ^a^	*F*_w_ (*p*) ^b^	× Time, *F*_in_^c^			× Time, *F*_in_^c^			× Time, *F*_in_^c^		
	Mean (SD)		Mean (SD)		Mean (SD)				*F*	SE	*p* ^d^	*F*	SE	*p* ^e^	*F*	SE	*p* ^d^
**FVC (L)**							−0.2 (0.540)	0.4 (0.011) *	−0.061	0.163	0.712	0.048	0.176	0.788	−0.109	0.168	0.524
Baseline	3.3 (0.7)		3.4 (0.9)		3.5 (1.2)												
12 weeks	3.7 (0.5)		3.6 (0.9)		3.8 (1.3)												
**FEV** **_1_** **(L)**							−0.2 (0.431)	0.1 (0.280)	0.079	0.132	0.562	−0.048	0.171	0.782	0.126	0.151	0.415
Baseline	2.6 (0.6)		2.7 (0.7)		2.8 (0.9)												
12 weeks	2.9 (0.4)		3.0 (0.8)		3.0 (1.0)												
**FEV** **_1/_** **FVC**							−0.02 (0.505)	−0.05 (0.044) *	0.045	0.021	0.043 *	−0.010	0.031	0.746	0.055	0.030	0.075
Baseline	0.8 (0.07)		0.8 (0.09)		0.8 (0.06)												
12 weeks	0.8 (0.1)		0.8 (0.04)		0.8 (0.03)												
**VO_2_** **max (ml/kg/min)**							−2.7 (0.232)	−1.8 (0.140)	5.30	1.26	0.001 *	2.59	1.67	0.134	2.72	1.38	0.066
Baseline	22.0 (5.4)		23.3 (5.9)		24.7 (6.0)												
12 weeks	25.0 (4.8)		24.2 (7.9)		22.7 (6.5)												
**VO_2_ max predicted (%)**							−16.0 (0.075)	−6.7 (0.199)	21.6	5.76	0.002 *	11.3	7.17	0.129	10.3	5.53	0.081
Baseline	88.0 (22.0)		95.9 (21.1)		104.0 (25.2)												
12 weeks	100.8 (22.6)		96.0 (26.6)		96.4 (28.6)												
**Work (watts)**							−15.7 (0.137)	−1.7 (0.766)	22.5	5.61	0.001 *	21.7	7.88	0.012 *	0.845	7.32	0.910
Baseline	73.5 (17.9)		90.6 (35.7)		89.1 (24.7)												
12 weeks	94.7 (21.7)		89.3 (36.3)		87.7 (29.0)												
**AT (L/min)**							−0.06 (0.496)	0.03 (0.517)	0.165	0.059	0.011 *	0.175	0.052	0.004 *	−0.010	0.045	0.828
Baseline	0.77 (0.2)		0.81 (0.3)		0.83 (0.2)												
12 weeks	0.96 (0.2)		0.88 (0.3)		0.88 (0.2)												

Note: FVC, forced vital capacity; FEV_1_, forced expiratory volume in one second; VO_2_ max, maximal oxygen uptake; AT, anaerobic threshold. ^a^ *F*_b_: The *F* value of between groups comparison. ^b^ *F***_w_**: The *F* value of within pre- and post-test. ^c^ *F*_in_: The *F* value of the interaction of between groups and within pre- and post-test. ^d^ Reference group: Group (Control) × Time. ^e^ Reference group: Group (Comparison) × Time. * *p* < 0.05. The Bonferroni test was used for post-hoc comparison.

## Data Availability

No new data were created or analyzed in this study. Data sharing is not applicable to this article.
